# Ischemic Stroke as the First Manifestation of a Mitral Valve Papillary Fibroelastoma: A Case Report

**DOI:** 10.7759/cureus.105744

**Published:** 2026-03-24

**Authors:** Jessica Fidalgo, Carina Santos, Ivanna Ostapiuk, Jorcelio Claudio Vicente, Cristina Gamboa

**Affiliations:** 1 Department of Internal Medicine, Guarda Local Health Unit, Guarda, PRT; 2 Department of Cardiology, Guarda Local Health Unit, Guarda, PRT

**Keywords:** cardioembolism, ischemic stroke, mitral valve, papillary fibroelastoma, transesophageal echocardiography

## Abstract

Cardiac papillary fibroelastomas are rare benign primary cardiac tumors that predominantly involve the endocardium. Despite their benign histological nature, they have a high embolic potential and may present as ischemic stroke, making their identification particularly relevant in the context of cryptogenic stroke.

We report the case of a 52-year-old woman who presented to the emergency department with sudden-onset right-sided hemiplegia and dysarthria. Computed tomography angiography revealed occlusion of the M1 segment of the left middle cerebral artery, and she underwent intravenous thrombolysis followed by mechanical thrombectomy, with favorable clinical evolution. During the etiological workup, transthoracic echocardiography revealed an image suggestive of a mass on the posterior leaflet of the mitral valve, later confirmed by transesophageal echocardiography, raising suspicion of a papillary fibroelastoma. The patient subsequently underwent cardiac surgery, which confirmed the diagnosis.

The aim of this report is to describe a rare case of ischemic stroke in association with a mitral valve papillary fibroelastoma and to highlight the importance of comprehensive etiological investigation in patients with presumed cryptogenic stroke.

## Introduction

Cardiac papillary fibroelastomas are rare benign primary cardiac tumors, accounting for approximately 8% of all cardiac tumors and representing the second most common benign cardiac tumor after myxoma [1]. These tumors usually arise from the valvular endocardium, with the aortic and mitral valves accounting for more than 80% of reported cases [2]. Although benign, cardiac papillary fibroelastomas are frequently associated with embolic phenomena, particularly ischemic stroke and transient ischemic attacks [3-5].

The pathophysiology of embolic events is related to fragmentation of the papillary projections of the tumor or detachment of superficial thrombi that form on its highly mobile and avascular surface [3]. Lesions located on left-sided valves (aortic and mitral), particularly those with high mobility or attachment to the free edge of the valve leaflets, carry a higher risk of embolization [4,5].

Mitral valve papillary fibroelastomas are less common than those arising from the aortic valve but have been repeatedly implicated in cryptogenic ischemic stroke, especially in young patients without cardiovascular risk factors [6,7]. Diagnosis may be challenging, as these lesions may not be detected on transthoracic echocardiography. Transesophageal echocardiography (TEE) is the most sensitive modality for detecting small and highly mobile valvular masses [7].

Given their embolic potential, surgical excision is generally recommended in symptomatic patients or in asymptomatic individuals with highly mobile lesions [1,4]. Recognition of this rare but potentially treatable cause of stroke is therefore essential. Although rare, papillary fibroelastoma should be considered in the differential diagnosis of embolic stroke, particularly when routine etiological investigations are unrevealing. In this report, we describe a case of ischemic stroke as the first clinical presentation associated with a mitral valve papillary fibroelastoma, highlighting the diagnostic challenges and the role of multimodal imaging in establishing the diagnosis and guiding management.

## Case presentation

A 52-year-old woman with a medical history of hypothyroidism, dyslipidemia, and previous tobacco use presented with sudden-onset paresthesia of the right lower limb and dysarthria at approximately 11:00 a.m., rapidly progressing to right-sided hemiplegia within minutes. She arrived at the emergency department at 12:48 p.m., approximately 1 hour and 48 minutes after symptom onset. Neurological examination revealed right-sided hemiplegia, right upper motor neuron (UMN) facial palsy, and dysarthria, with a National Institutes of Health Stroke Scale (NIHSS) score of 9 [8].

**Figure 1:**
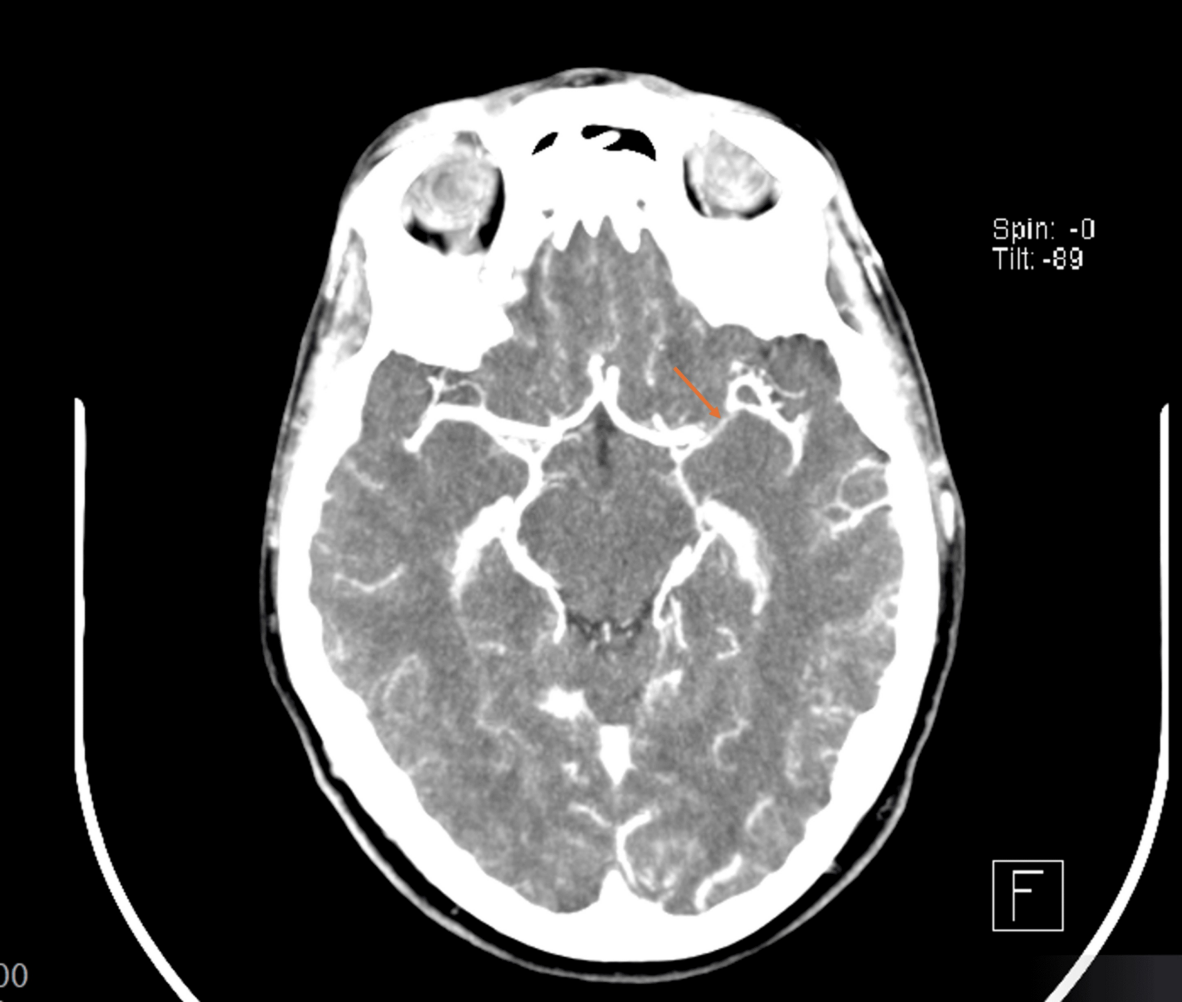
Intracranial CT angiography at initial admission CT demonstrating occlusion of the M1 segment of the left middle cerebral artery (arrow).

Initial non-contrast cranial computed tomography showed no acute abnormalities (ASPECTS 10). Computed tomography angiography revealed occlusion of the M1 segment of the left middle cerebral artery, with good collateral circulation and no significant stenosis of the supra-aortic trunks (Figure 1). The patient met criteria for intravenous thrombolysis according to current European Stroke Organisation guidelines [9,10]. Intravenous thrombolysis was administered approximately 2 hours and 20 minutes after symptom onset. The patient was then transferred to a tertiary care center, where mechanical thrombectomy was performed 6 hours and 27 minutes after symptom onset, achieving complete recanalization (TICI 3) at 6 hours and 40 minutes, without complications.

**Figure 2:**
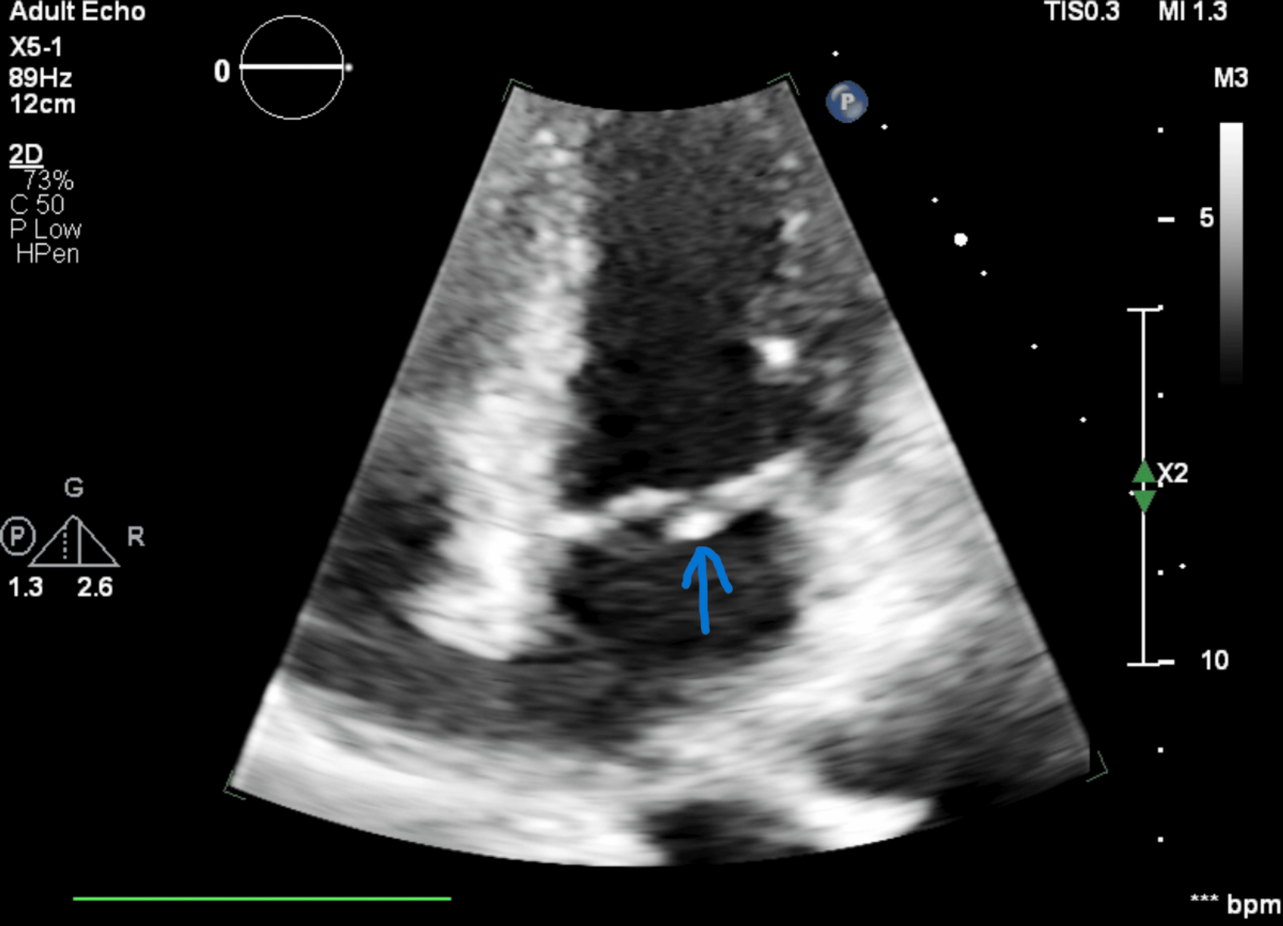
Transthoracic echocardiography Two-dimensional transthoracic echocardiographic image demonstrating a rounded, well-defined echogenic mass attached to the posterior leaflet of the mitral valve (arrow), raising suspicion of a valvular tumor.

She was admitted to the stroke unit. As part of the etiological investigation, carotid and vertebral Doppler ultrasonography excluded hemodynamically significant stenosis. Although 24-hour Holter monitoring did not reveal atrial fibrillation, the limited sensitivity of short-term monitoring for detecting paroxysmal atrial fibrillation after ischemic stroke is well recognized. Extended cardiac monitoring (up to 7-30 days) may increase detection rates. Transthoracic echocardiography with Doppler study showed mild mitral valve prolapse and a rounded, well-defined mass located on the posterior leaflet of the mitral valve (Figure 2), raising suspicion of a valvular tumor.

**Figure 3:**
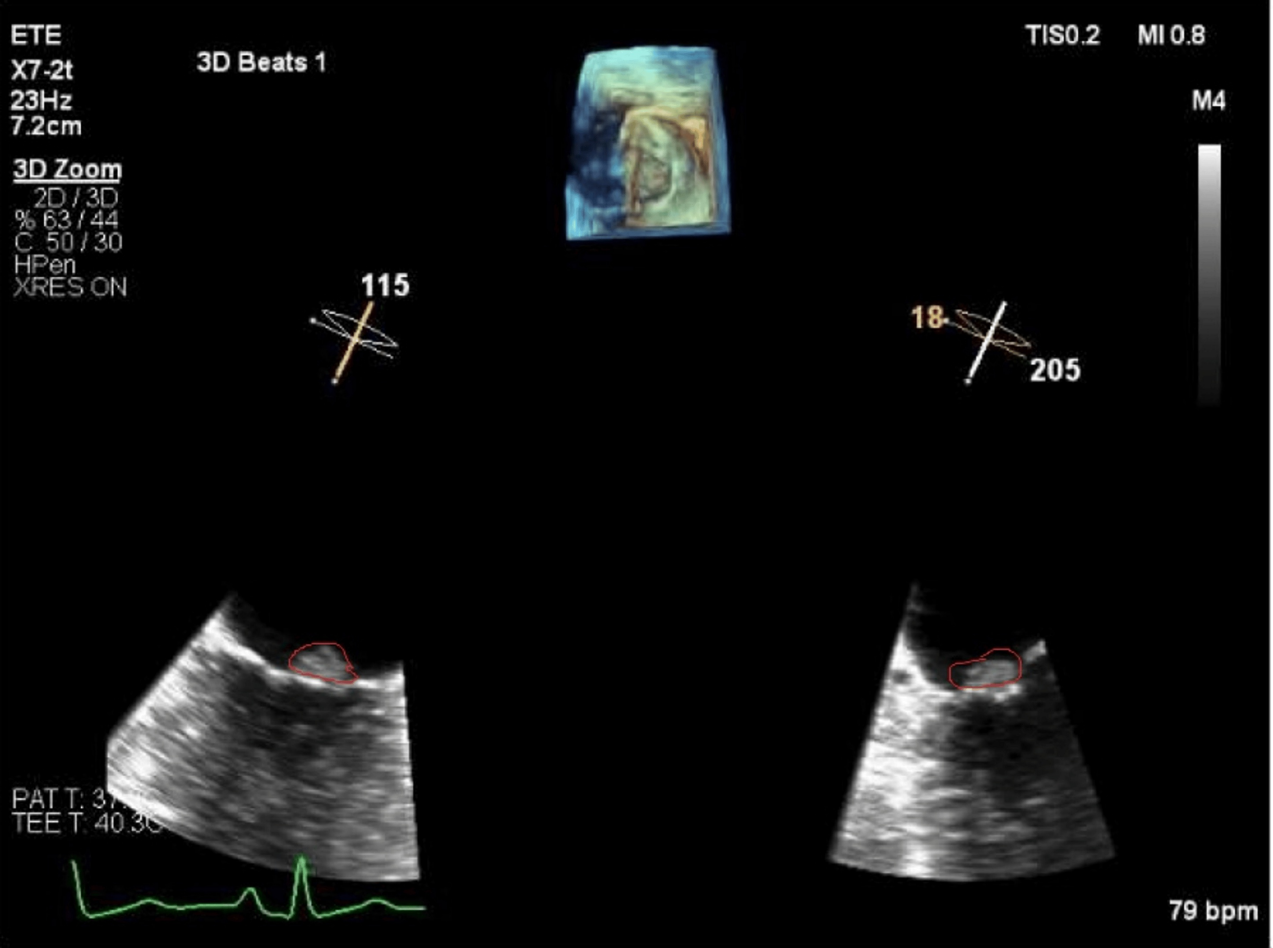
Three-dimensional transesophageal echocardiography (3D TEE) Image showing a rounded lesion attached to the atrial side of the posterior leaflet of the mitral valve.

Subsequent transesophageal echocardiography demonstrated a rounded mass with regular margins attached to the posterior leaflet of the mitral valve, suggestive of a papillary fibroelastoma (Figure 3).

**Figure 4:**
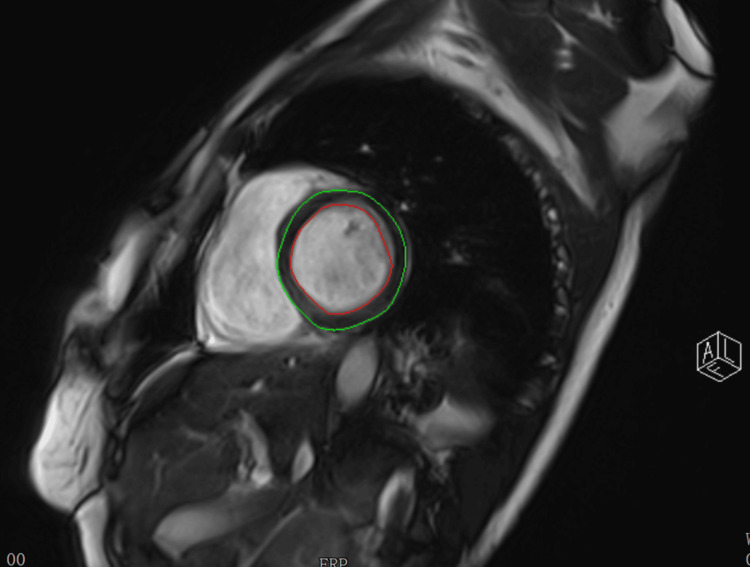
Cine segmented three-chamber cardiac magnetic resonance Image demonstrating a rounded nodular lesion (10 × 7 mm) arising from the posterior leaflet of the mitral valve, projecting toward the left atrium (circled). The lesion shows well-defined margins and imaging characteristics compatible with papillary fibroelastoma.

Cardiac magnetic resonance imaging further identified a 10 × 7 mm nodular lesion arising from the posterior mitral leaflet with late gadolinium enhancement, compatible with papillary fibroelastoma (Figure 4). Ventricular size and systolic function were preserved, the atria were not dilated, and no intracardiac thrombi were detected. No alternative embolic source was identified during the etiological investigation.

The patient showed favorable clinical evolution during hospitalization. At discharge, she had mild right brachial-predominant hemiparesis and perceptible dysarthria (NIHSS 4). Her CHA₂DS₂-VASc score was 3 (female sex and prior stroke). She was discharged on oral anticoagulation with edoxaban 60 mg once daily and referred for motor rehabilitation in a long-term care facility.

Six months later, the patient underwent cardiac surgery with excision of the mitral valve tumor. No interval echocardiographic studies documenting progression of the lesion were available prior to surgery. Histopathological examination of the excised mass confirmed the diagnosis of papillary fibroelastoma.

## Discussion

Cardiac papillary fibroelastomas, although histologically benign, are clinically significant due to their well-established association with embolic events, particularly ischemic stroke. Large series and systematic analyses have demonstrated that cardiac fibroelastomas are among the most emboligenic benign cardiac tumors, with neurological events representing one of the most frequent clinical manifestations [1,3]. Advances in cardiac imaging have increased detection rates; however, fibroelastomas remain an underrecognized cause of cryptogenic stroke, especially in patients without conventional cardiovascular risk factors [3,6].

Although a papillary fibroelastoma was identified and represents a plausible embolic source, causality cannot be definitively established. Other potential causes of ischemic stroke, including paroxysmal atrial fibrillation and occult embolic sources, cannot be entirely excluded, particularly given the absence of prolonged cardiac monitoring.

In the present case, a mitral valve papillary fibroelastoma was identified during the etiological investigation of an acute ischemic stroke involving the left middle cerebral artery territory. This presentation is consistent with prior reports showing that stroke is the most common initial manifestation of symptomatic PFEs, often involving large-vessel occlusion of the anterior circulation [3,6]. Although PFEs most frequently arise from the aortic valve, mitral valve involvement has been repeatedly implicated in cerebrovascular events, particularly in younger patients [2,7].

Mitral valve papillary fibroelastomas appear to confer a particularly high embolic risk. Observational studies and pooled analyses suggest that left-sided PFEs account for the majority of embolic complications, with the mitral valve involved in up to 35-40% of cases presenting with stroke [1,3]. While the anterior mitral leaflet is more commonly affected, posterior leaflet involvement, as observed in our patient, has also been associated with embolic phenomena, particularly when lesions are mobile or pedunculated [2,5].

The pathophysiology of embolization in fibroelastomas is incompletely understood. Two main mechanisms have been proposed: fragmentation of the friable papillary fronds and embolization of thrombotic material forming on the tumor’s irregular, avascular surface [1,5]. Histopathological studies and reports of tumor fragments retrieved during mechanical thrombectomy support the hypothesis that embolization may occur despite antithrombotic therapy, highlighting the intrinsic embolic potential of these lesions [3,6].

Diagnosis of papillary fibroelastoma can be challenging, particularly when lesions are small or highly mobile. Transthoracic echocardiography is often the first-line imaging modality but may fail to detect lesions measuring only a few millimeters. Transesophageal echocardiography remains the most sensitive diagnostic tool, providing superior spatial resolution and allowing accurate characterization of tumor attachment, mobility, and morphology [2,5]. In large series, up to 10-15% of fibroelastomas were detected exclusively by transesophageal echocardiography after inconclusive transthoracic studies [2,5]. Cardiac magnetic resonance imaging may provide complementary information, particularly for tissue characterization and differentiation from thrombus or vegetation, although its role is secondary to echocardiography in highly mobile valvular lesions [7]. Unlike cardiac myxomas, which may produce interleukin-6 and cause systemic inflammatory manifestations with elevated erythrocyte sedimentation rate (ESR) and C-reactive protein (CRP), papillary fibroelastomas are small avascular endocardial tumors and typically do not trigger a systemic inflammatory response; therefore, inflammatory laboratory markers are not helpful for their diagnosis.

Management of papillary fibroelastomas remains debated in asymptomatic patients; however, there is broad consensus that surgical excision is indicated in symptomatic patients and in those with left-sided, mobile tumors due to the high risk of embolization [1,4]. Surgical resection is generally curative, associated with low perioperative morbidity, and allows valve-sparing procedures in the majority of cases [4,7]. Long-term follow-up studies have demonstrated a marked reduction in recurrent cerebrovascular events and excellent prognosis after tumor excision, with recurrence being exceedingly rare [1,3,4].

In the present case, the patient underwent surgical excision six months after the index stroke, with histopathological confirmation of papillary fibroelastoma and no reported recurrence. This favorable outcome reinforces the importance of recognizing papillary fibroelastoma as a rare but potentially curable cause of ischemic stroke. Early identification of papillary fibroelastomas is essential, particularly in patients presenting with ischemic stroke [6]. Surgical excision represents definitive treatment and significantly reduces the risk of recurrent embolic events [1,3,4].

## Conclusions

Despite their rarity and benign histological nature, cardiac papillary fibroelastomas may represent an important cause of ischemic stroke. This case highlights the importance of a thorough etiological investigation in ischemic stroke, particularly when common causes are excluded. Identification of valvular masses, especially those involving the mitral valve, should prompt targeted imaging evaluation, given the potential benefit of definitive surgical management. Early recognition of this entity may reduce the risk of recurrence and improve patient prognosis.
